# Adamantane-1-ammonium benzoate

**DOI:** 10.1107/S1600536809036903

**Published:** 2009-10-17

**Authors:** Wen-Ni Zheng, Bo Wang

**Affiliations:** a Ordered Matter Science Research Center, College of Chemistry and Chemical, Engineering, Southeast University, Nanjing 211189, People’s Republic of China

## Abstract

In the title molecular salt, C_10_H_15_NH_3_^+^·C_7_H_5_O_2_^−^, both carboxyl O atoms act as acceptors for strong N—H⋯O inter­molecular hydrogen-bond inter­actions with the ammonium group in the cation, generating infinite chains along the *b* axis. A weak C—H⋯π inter­action is also present.

## Related literature

For related structures, see: Tukada & Mochizuki (2003 ▶); Zhao *et al.* (2003 ▶); He & Wen (2006 ▶). For puckering parameters, see: Cremer & Pople (1975 ▶). 
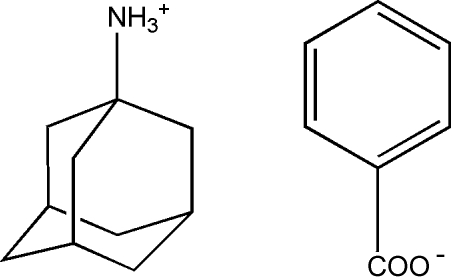


## Experimental

### 

#### Crystal data

C_10_H_18_N^+^·C_7_H_5_O_2_^−^
*M* *_r_* = 273.36
Monoclinic, 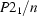

*a* = 10.918 (2) Å
*b* = 6.5664 (13) Å
*c* = 21.197 (4) Å
β = 100.07 (3)°
*V* = 1496.3 (5) Å^3^
*Z* = 4
Mo *K*α radiation
μ = 0.08 mm^−1^
*T* = 298 K
0.20 × 0.20 × 0.20 mm

#### Data collection

Rigaku SCXmini diffractometer
Absorption correction: multi-scan (*CrystalClear*; Rigaku, 2005 ▶) *T*_min_ = 0.774, *T*_max_ = 1.000
15027 measured reflections
3437 independent reflections
2453 reflections with *I* > 2σ(*I*)
*R*_int_ = 0.043

#### Refinement

*R*[*F* ^2^ > 2σ(*F* ^2^)] = 0.055
*wR*(*F* ^2^) = 0.147
*S* = 1.04
3437 reflections
181 parameters
H-atom parameters constrained
Δρ_max_ = 0.20 e Å^−3^
Δρ_min_ = −0.26 e Å^−3^

### 

Data collection: *CrystalClear* (Rigaku 2005 ▶); cell refinement: *CrystalClear*; data reduction: *CrystalClear*; program(s) used to solve structure: *SHELXS97* (Sheldrick, 2008 ▶); program(s) used to refine structure: *SHELXL97* (Sheldrick, 2008 ▶); molecular graphics: *SHELXTL* (Sheldrick, 2008 ▶); software used to prepare material for publication: *PRPKAPPA* (Ferguson, 1999 ▶).

## Supplementary Material

Crystal structure: contains datablocks I, New_Global_Publ_Block. DOI: 10.1107/S1600536809036903/jj2008sup1.cif

Structure factors: contains datablocks I. DOI: 10.1107/S1600536809036903/jj2008Isup2.hkl

Additional supplementary materials:  crystallographic information; 3D view; checkCIF report

## Figures and Tables

**Table 1:** Hydrogen-bond geometry (Å, °)

*D*—H⋯*A*	*D*—H	H⋯*A*	*D*⋯*A*	*D*—H⋯*A*
N1—H1*A*⋯O1^i^	0.89	1.83	2.7134 (17)	176
N1—H1*B*⋯O2^ii^	0.89	1.90	2.7840 (18)	173
N1—H1*C*⋯O2	0.89	1.92	2.7915 (18)	166
C16—H16*A*⋯*Cg*1^iii^	0.97	2.74	3.702 (2)	174

Symmetry codes: (i) 
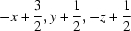
; (ii) 
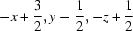
; (iii) 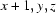. *Cg*1 is the centroid of the C2–C7 ring.

**Fig. 1.**
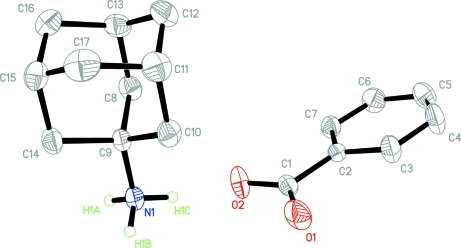
The molecular structure of the title compound, with the atomic numbering scheme and displacement ellipsoids drawn at the 30% probability level. All H atoms except those on the N atom have been omitted for clarity.

**Fig. 2.**
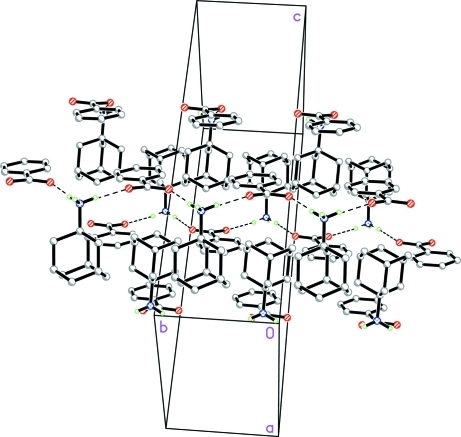
A view of the crystal packing of the title compound. Dashed lines indicate N–H···O hydrogen bonds which form infinite, one-dimensional chains along the b axis of the unit cell. H atoms not involved in hydrogen bonding have been omitted for clarity.

## supplementary crystallographic information

### Comment

Owing to its highly symmetrical and stable structure, adamantane and its derivatives have generated much interest in the past and continue to be actively studied as evidenced by the large number of compounds containing amantadine that have been synthesized (Tukada & Mochizuki, 2003; Zhao *et al.*, 2003; He & Wen, 2006). Here we report the synthesis and crystal structure of the title compound,(I), C_10_H_15_NH_3_^+^ . C_7_H_5_O_2_^-^, a salt obtained from the reaction of adamantane-1-ammonium hydrochloride and sodium benzoate (Fig. 1).

The adamantane-1-ammonium cation contains four 6-membered rings in a cage-like structure each in a slightly distorted boat conformation and with a protonated *N* atom at the 1-position. Puckering parameters (Cremer & Pople, 1975) Q, θ and φ are for rings 1–4 [(1) 0.630 (2) Å, 1.48 (18)°,272 (54)°; (2) 0.6247 (19)Å,178.36 (17)°, 251 (423)°; (3) 0.621 (4)Å,0.67 (18)°, 240 (54)°; (4)0.6207 (19)Å, 0.55 (18)°, 218 (39)°] where (1) = C7–C9/C13–C15, (2) = C7/C8/C10/C11/C16/C15, (3) = C8/C9/C13/C12/C11/C10 and (4) = C11–C16. C—C distances range from 1.518 (2)Å to 1.531 (3)Å and C—C—C angles range from 108.93 (13)° to 109.91 (13)°, while the exocyclic C—N bond length is 1.4924 (19)Å. These values are similar to that observed in adamantane-1-ammonium 2-nitrobenzoate (C—C = 1.5254 (18)Å to 1.532 (2)Å, C—C—C 109.06 (13)° to 109.84 (11)°, C—N = 1.4967 (18)Å (He & Wen, 2006). Both the negativly charged and neutral oxygen atoms in the benzoate anion are involved in strong N—H···O intermolecular hydrogen bond interactions with the ammonium cation group generating infinite one-dimensional chains along the *b* axis of the unit cell (Fig. 2, Table 1). In addition, weak π-ring C16–H16A···Cg1 interactions exist which contribute to crystal stability (Cg1 is the center of gravity of ring 1).

### Experimental

A mixture of adamantane-1- ammonium hydrochloride (10 mmol), sodiumbenzoate (10 mmol) and methanol (50 ml) was stirred in a beaker. There were many solid powders produced and the solution was filtered. Colorless single crystals of the title compound suitable for X-ray analysis were obtained by slow evaporation of the solvents over a period of 20 h.

### Refinement

Positional parameters of all the H atoms were calculated geometrically (aromatic C–H = 0.93 A°, aliphatic C–H = 0.97 A°) & N–H = 0.89Å) and were allowed to ride on the C,N atoms to which they are bonded, with *U*_iso_(H) = 1.2-1.5*U*_eq_(C,N).

### Crystal data

**Table:**

C_10_H_18_N^+^·C_7_H_5_O_2_^−^	*F*(000) = 592
*M**_r_* = 273.36	*D*_x_ = 1.214 Mg m^−^^3^
Monoclinic, *P*2_1_/*n*	Mo *K*α radiation, λ = 0.71073 Å
Hall symbol: -P 2yn	Cell parameters from 12490 reflections
*a* = 10.918 (2) Å	θ = 3.2–27.7°
*b* = 6.5664 (13) Å	µ = 0.08 mm^−^^1^
*c* = 21.197 (4) Å	*T* = 298 K
β = 100.07 (3)°	Prism, colourless
*V* = 1496.3 (5) Å^3^	0.20 × 0.20 × 0.20 mm
*Z* = 4	

### Data collection

**Table:**

Rigaku SCXmini diffractometer	3437 independent reflections
Radiation source: fine-focus sealed tube	2453 reflections with *I* > 2σ(*I*)
graphite	*R*_int_ = 0.043
Detector resolution: 13.6612 pixels mm^-1^	θ_max_ = 27.5°, θ_min_ = 3.2°
CCD_Profile_fitting scans	*h* = −14→14
Absorption correction: multi-scan (*CrystalClear*; Rigaku, 2005)	*k* = −8→8
*T*_min_ = 0.774, *T*_max_ = 1.000	*l* = −27→26
15027 measured reflections	

### Refinement

**Table:**

Refinement on *F*^2^	Primary atom site location: structure-invariant direct methods
Least-squares matrix: full	Secondary atom site location: difference Fourier map
*R*[*F*^2^ > 2σ(*F*^2^)] = 0.055	Hydrogen site location: inferred from neighbouring sites
*wR*(*F*^2^) = 0.147	H-atom parameters constrained
*S* = 1.03	*w* = 1/[σ^2^(*F*_o_^2^) + (0.0654*P*)^2^ + 0.3147*P*] where *P* = (*F*_o_^2^ + 2*F*_c_^2^)/3
3437 reflections	(Δ/σ)_max_ < 0.001
181 parameters	Δρ_max_ = 0.20 e Å^−^^3^
0 restraints	Δρ_min_ = −0.26 e Å^−^^3^

### Special details

**Table:**

Geometry. All esds (except the esd in the dihedral angle between two l.s. planes) are estimated using the full covariance matrix. The cell esds are taken into account individually in the estimation of esds in distances, angles and torsion angles; correlations between esds in cell parameters are only used when they are defined by crystal symmetry. An approximate (isotropic) treatment of cell esds is used for estimating esds involving l.s. planes.
Refinement. Refinement of *F*^2^ against ALL reflections. The weighted *R*-factor *wR* and goodness of fit *S* are based on *F*^2^, conventional *R*-factors *R* are based on *F*, with *F* set to zero for negative *F*^2^. The threshold expression of *F*^2^ > σ(*F*^2^) is used only for calculating *R*-factors(gt) *etc*. and is not relevant to the choice of reflections for refinement. *R*-factors based on *F*^2^ are statistically about twice as large as those based on *F*, and *R*- factors based on ALL data will be even larger.

### Fractional atomic coordinates and isotropic or equivalent isotropic displacement parameters (Å^2^)

**Table:**

	*x*	*y*	*z*	*U*_iso_*/*U*_eq_	
C1	0.56217 (14)	0.6892 (3)	0.15342 (7)	0.0405 (4)	
C2	0.47020 (13)	0.7614 (2)	0.09647 (7)	0.0365 (3)	
C3	0.41146 (17)	0.6184 (3)	0.05392 (8)	0.0551 (5)	
H3A	0.4282	0.4807	0.0612	0.066*	
C4	0.3278 (2)	0.6789 (3)	0.00041 (9)	0.0718 (6)	
H4A	0.2899	0.5820	−0.0286	0.086*	
C5	0.30094 (19)	0.8799 (4)	−0.00983 (9)	0.0707 (6)	
H5A	0.2439	0.9201	−0.0455	0.085*	
C6	0.35764 (18)	1.0225 (3)	0.03223 (9)	0.0651 (5)	
H6A	0.3385	1.1596	0.0253	0.078*	
C7	0.44324 (15)	0.9646 (3)	0.08504 (8)	0.0481 (4)	
H7A	0.4828	1.0629	0.1130	0.058*	
C8	0.93359 (16)	0.8861 (2)	0.16653 (8)	0.0463 (4)	
H8A	0.9568	1.0003	0.1952	0.056*	
H8B	0.8483	0.9057	0.1452	0.056*	
C9	0.94432 (13)	0.6891 (2)	0.20453 (7)	0.0347 (3)	
C10	0.90627 (16)	0.5096 (2)	0.15964 (8)	0.0472 (4)	
H10A	0.9123	0.3838	0.1840	0.057*	
H10B	0.8207	0.5260	0.1383	0.057*	
C11	0.99218 (19)	0.5007 (3)	0.11014 (9)	0.0591 (5)	
H11A	0.9681	0.3859	0.0811	0.071*	
C12	0.98200 (19)	0.6977 (3)	0.07187 (9)	0.0633 (5)	
H12A	0.8972	0.7158	0.0496	0.076*	
H12B	1.0360	0.6915	0.0401	0.076*	
C13	1.01933 (18)	0.8766 (3)	0.11689 (9)	0.0548 (5)	
H13A	1.0126	1.0035	0.0922	0.066*	
C14	1.07795 (14)	0.6598 (3)	0.23908 (8)	0.0474 (4)	
H14A	1.0846	0.5347	0.2638	0.057*	
H14B	1.1021	0.7723	0.2683	0.057*	
C15	1.16369 (16)	0.6503 (3)	0.18941 (9)	0.0596 (5)	
H15A	1.2497	0.6317	0.2113	0.071*	
C16	1.15367 (17)	0.8477 (3)	0.15120 (10)	0.0628 (5)	
H16A	1.2089	0.8428	0.1200	0.075*	
H16B	1.1783	0.9616	0.1797	0.075*	
C17	1.1262 (2)	0.4714 (3)	0.14452 (11)	0.0689 (6)	
H17A	1.1330	0.3455	0.1688	0.083*	
H17B	1.1813	0.4629	0.1134	0.083*	
N1	0.85951 (11)	0.69816 (19)	0.25257 (6)	0.0393 (3)	
H1A	0.8810	0.8025	0.2790	0.059*	
H1B	0.8654	0.5828	0.2749	0.059*	
H1C	0.7815	0.7146	0.2324	0.059*	
O1	0.57302 (14)	0.5030 (2)	0.16200 (6)	0.0744 (5)	
O2	0.62592 (11)	0.81896 (19)	0.18784 (6)	0.0594 (4)	

### Atomic displacement parameters (Å^2^)

**Table:**

	*U*^11^	*U*^22^	*U*^33^	*U*^12^	*U*^13^	*U*^23^
C1	0.0380 (8)	0.0485 (9)	0.0338 (8)	0.0034 (7)	0.0030 (6)	−0.0005 (7)
C2	0.0332 (7)	0.0430 (8)	0.0325 (7)	−0.0025 (6)	0.0039 (6)	0.0033 (6)
C3	0.0573 (10)	0.0494 (10)	0.0514 (10)	−0.0022 (8)	−0.0102 (8)	−0.0026 (8)
C4	0.0713 (13)	0.0782 (15)	0.0541 (11)	−0.0070 (11)	−0.0218 (10)	−0.0098 (10)
C5	0.0622 (12)	0.0890 (16)	0.0518 (11)	0.0029 (11)	−0.0148 (9)	0.0201 (11)
C6	0.0635 (12)	0.0581 (12)	0.0670 (12)	0.0025 (9)	−0.0073 (10)	0.0234 (10)
C7	0.0473 (9)	0.0454 (9)	0.0482 (9)	−0.0030 (7)	−0.0012 (7)	0.0045 (7)
C8	0.0529 (9)	0.0362 (9)	0.0467 (9)	0.0043 (7)	−0.0002 (7)	0.0037 (7)
C9	0.0346 (7)	0.0325 (7)	0.0343 (7)	−0.0002 (6)	−0.0019 (6)	−0.0008 (6)
C10	0.0521 (9)	0.0385 (9)	0.0493 (9)	−0.0068 (7)	0.0046 (8)	−0.0090 (7)
C11	0.0723 (12)	0.0516 (11)	0.0546 (11)	−0.0037 (9)	0.0137 (9)	−0.0182 (8)
C12	0.0675 (12)	0.0823 (14)	0.0398 (9)	−0.0007 (10)	0.0083 (9)	−0.0031 (9)
C13	0.0648 (11)	0.0487 (10)	0.0507 (10)	−0.0016 (8)	0.0092 (9)	0.0150 (8)
C14	0.0391 (8)	0.0527 (10)	0.0461 (9)	0.0016 (7)	−0.0045 (7)	0.0046 (7)
C15	0.0375 (8)	0.0759 (13)	0.0635 (11)	0.0061 (8)	0.0036 (8)	0.0076 (10)
C16	0.0528 (10)	0.0718 (13)	0.0651 (12)	−0.0150 (9)	0.0134 (9)	0.0019 (10)
C17	0.0717 (13)	0.0601 (12)	0.0810 (14)	0.0191 (10)	0.0297 (11)	0.0009 (10)
N1	0.0371 (6)	0.0397 (7)	0.0375 (7)	0.0005 (5)	−0.0030 (5)	−0.0023 (5)
O1	0.0912 (11)	0.0536 (9)	0.0663 (9)	0.0002 (7)	−0.0200 (8)	0.0209 (6)
O2	0.0484 (7)	0.0625 (8)	0.0575 (7)	0.0142 (6)	−0.0177 (6)	−0.0197 (6)

### Geometric parameters (Å, °)

**Table:**

C1—O1	1.238 (2)	C10—H10B	0.9700
C1—O2	1.2512 (19)	C11—C12	1.520 (3)
C1—C2	1.505 (2)	C11—C17	1.528 (3)
C2—C7	1.378 (2)	C11—H11A	0.9800
C2—C3	1.380 (2)	C12—C13	1.523 (3)
C3—C4	1.384 (3)	C12—H12A	0.9700
C3—H3A	0.9300	C12—H12B	0.9700
C4—C5	1.361 (3)	C13—C16	1.531 (3)
C4—H4A	0.9300	C13—H13A	0.9800
C5—C6	1.365 (3)	C14—C15	1.528 (2)
C5—H5A	0.9300	C14—H14A	0.9700
C6—C7	1.380 (2)	C14—H14B	0.9700
C6—H6A	0.9300	C15—C17	1.522 (3)
C7—H7A	0.9300	C15—C16	1.522 (3)
C8—C9	1.518 (2)	C15—H15A	0.9800
C8—C13	1.527 (2)	C16—H16A	0.9700
C8—H8A	0.9700	C16—H16B	0.9700
C8—H8B	0.9700	C17—H17A	0.9700
C9—N1	1.4925 (19)	C17—H17B	0.9700
C9—C14	1.526 (2)	N1—H1A	0.8900
C9—C10	1.526 (2)	N1—H1B	0.8900
C10—C11	1.526 (2)	N1—H1C	0.8900
C10—H10A	0.9700		
			
O1—C1—O2	123.88 (15)	C17—C11—H11A	109.4
O1—C1—C2	117.52 (14)	C11—C12—C13	109.58 (14)
O2—C1—C2	118.55 (15)	C11—C12—H12A	109.8
C7—C2—C3	118.90 (15)	C13—C12—H12A	109.8
C7—C2—C1	122.54 (14)	C11—C12—H12B	109.8
C3—C2—C1	118.55 (14)	C13—C12—H12B	109.8
C2—C3—C4	120.31 (18)	H12A—C12—H12B	108.2
C2—C3—H3A	119.8	C12—C13—C8	109.53 (15)
C4—C3—H3A	119.8	C12—C13—C16	109.35 (16)
C5—C4—C3	120.12 (18)	C8—C13—C16	109.25 (15)
C5—C4—H4A	119.9	C12—C13—H13A	109.6
C3—C4—H4A	119.9	C8—C13—H13A	109.6
C4—C5—C6	120.05 (17)	C16—C13—H13A	109.6
C4—C5—H5A	120.0	C9—C14—C15	108.92 (13)
C6—C5—H5A	120.0	C9—C14—H14A	109.9
C5—C6—C7	120.41 (18)	C15—C14—H14A	109.9
C5—C6—H6A	119.8	C9—C14—H14B	109.9
C7—C6—H6A	119.8	C15—C14—H14B	109.9
C2—C7—C6	120.18 (16)	H14A—C14—H14B	108.3
C2—C7—H7A	119.9	C17—C15—C16	109.76 (16)
C6—C7—H7A	119.9	C17—C15—C14	109.41 (15)
C9—C8—C13	109.32 (13)	C16—C15—C14	109.63 (15)
C9—C8—H8A	109.8	C17—C15—H15A	109.3
C13—C8—H8A	109.8	C16—C15—H15A	109.3
C9—C8—H8B	109.8	C14—C15—H15A	109.3
C13—C8—H8B	109.8	C15—C16—C13	109.38 (15)
H8A—C8—H8B	108.3	C15—C16—H16A	109.8
N1—C9—C8	109.18 (12)	C13—C16—H16A	109.8
N1—C9—C14	109.45 (12)	C15—C16—H16B	109.8
C8—C9—C14	109.91 (13)	C13—C16—H16B	109.8
N1—C9—C10	108.82 (12)	H16A—C16—H16B	108.2
C8—C9—C10	109.89 (12)	C15—C17—C11	109.36 (15)
C14—C9—C10	109.58 (13)	C15—C17—H17A	109.8
C11—C10—C9	108.97 (13)	C11—C17—H17A	109.8
C11—C10—H10A	109.9	C15—C17—H17B	109.8
C9—C10—H10A	109.9	C11—C17—H17B	109.8
C11—C10—H10B	109.9	H17A—C17—H17B	108.3
C9—C10—H10B	109.9	C9—N1—H1A	109.5
H10A—C10—H10B	108.3	C9—N1—H1B	109.5
C12—C11—C10	109.76 (15)	H1A—N1—H1B	109.5
C12—C11—C17	109.64 (17)	C9—N1—H1C	109.5
C10—C11—C17	109.22 (15)	H1A—N1—H1C	109.5
C12—C11—H11A	109.4	H1B—N1—H1C	109.5
C10—C11—H11A	109.4		

### Hydrogen-bond geometry (Å, °)

**Table:**

*D*—H···*A*	*D*—H	H···*A*	*D*···*A*	*D*—H···*A*
N1—H1A···O1^i^	0.89	1.83	2.7134 (17)	176
N1—H1B···O2^ii^	0.89	1.90	2.7840 (18)	173
N1—H1C···O2	0.89	1.92	2.7915 (18)	166
C16—H16A···Cg1^iii^	0.97	2.74	3.702 (2)	174

Symmetry codes: (i) −*x*+3/2, *y*+1/2, −*z*+1/2; (ii) −*x*+3/2, *y*−1/2, −*z*+1/2; (iii) *x*+1, *y*, *z*.
